# Zika Virus NS1 Drives Tunneling Nanotube Formation for Mitochondrial Transfer, Enhanced Survival, Interferon Evasion, and Stealth Transmission in Trophoblasts

**DOI:** 10.21203/rs.3.rs-3674059/v1

**Published:** 2023-12-06

**Authors:** Indira Mysorekar, Rafael Michita, Long Tran, Steven Bark, Deepak Kumar, Shay Toner, Joyce Jose, Anoop Narayanan

**Affiliations:** Baylor College of Medicine; Baylor College of Medicine; Baylor College of Medicine; Baylor College of Medicine; Baylor College of Medicine; Pennsylvania State University; The Pennsylvania State University; Penn State University

## Abstract

Zika virus (ZIKV) infection continues to pose a significant public health concern due to limited available preventive measures and treatments. ZIKV is unique among flaviviruses in its vertical transmission capacity (i.e., transmission from mother to fetus) yet the underlying mechanisms remain incompletely understood. Here, we show that both African and Asian lineages of ZIKV induce tunneling nanotubes (TNTs) in placental trophoblasts and multiple other mammalian cell types. Amongst investigated flaviviruses, only ZIKV strains trigger TNTs. We show that ZIKV-induced TNTs facilitate transfer of viral particles, proteins, and RNA to neighboring uninfected cells. ZIKV TNT formation is driven exclusively via its non-structural protein 1 (NS1); specifically, the N-terminal region (50 aa) of membrane-bound NS1 is necessary and sufficient for triggering TNT formation in host cells. Using affinity purification-mass spectrometry of cells infected with wild-type NS1 or non-TNT forming NS1 (pNS1ΔTNT) proteins, we found mitochondrial proteins are dominant NS1-interacting partners, consistent with the elevated mitochondrial mass we observed in infected trophoblasts. We demonstrate that mitochondria are siphoned via TNTs from healthy to ZIKV-infected cells, both homotypically and heterotypically, and inhibition of mitochondrial respiration reduced viral replication in trophoblast cells. Finally, ZIKV strains lacking TNT capabilities due to mutant NS1 elicited a robust antiviral IFN-λ 1/2/3 response, indicating ZIKV’s TNT-mediated trafficking also allows ZIKV cell-cell transmission that is camouflaged from host defenses. Together, our findings identify a new stealth mechanism that ZIKV employs for intercellular spread among placental trophoblasts, evasion of antiviral interferon response, and the hijacking of mitochondria to augment its propagation and survival. Discerning the mechanisms of ZIKV intercellular strategies offers a basis for novel therapeutic developments targeting these interactions to limit its dissemination.

## Introduction

Zika virus (ZIKV) is a mosquito-borne positive-strand RNA virus within the *Flaviviridae* family. Following a period of sporadic infections in Africa and Asia, ZIKV began rapidly spreading in the Pacific islands (2007–2013)^1,2^ before reaching epidemic proportions in the Americas (2015–2018)^3^, with estimates as high as 130 million total cases by 2018^4^. While the adult infection is typically mild or asymptomatic (~80%), ZIKV infection can lead to neurological disorders and fetal abnormalities such as microcephaly and fetal demise, collectively known as congenital Zika syndrome^5,6^. ZIKV s propensity for horizontal and vertical transmission^7–10^, and its ability to traverse blood-tissue barriers, including the blood-placental barrier^11–13^, are unique among *Flaviviridae*^6,14^. Murine studies from our group and others have shown that ZIKV can infect fetal trophoblasts and endothelial cells of the placenta, which form the primary barrier between the maternal and fetal circulations, leading to ZIKV entry into fetal circulation^11^. Other studies show ZIKV has broad tropism in the human placenta^15–20^, infecting cytotrophoblasts (CTBs), syncytiotrophoblasts (STBs), extravillous trophoblasts (EVTs), endothelial cells, and fetal macrophages in the intervillous space, possibly allowing the virus to cross the protective barrier. Different trophoblasts show differential permissiveness for ZIKV. For example, STBs derived from primary human trophoblasts (PHTs) are more resistant due to interferon (IFN)-λ response. At the same time, CTBs and EVTs, represented by cell lines JEG-3 and HTR-8, respectively, appear susceptible to ZIKV infection^11,16^. Antiviral IFN response, mainly mediated by type-III (IFN-λ), seems to confer protection against ZIKV^16,21,22^.

The flavivirus positive-strand RNA genome encodes three structural proteins (C, prM, and E) and seven nonstructural proteins (NS1, NS2A, NS2B, NS3, NS4A, NS4B, and NS5). ZIKV nonstructural proteins participate in viral replication, assembly, and hijack host biological processes^23–27^. NS1 is a glycosylated, membrane-associated non-structural protein of ZIKV existing as a dimer in the endoplasmic reticulum (ER)^28,29^, where it is essential for viral RNA replication^29^ or as a hexamer secreted from the infected cells with diverse roles in immune evasion and pathogenesis^30^. ZIKV NS1 shares structural similarities with NS1 proteins of other flaviviruses like dengue virus (DENV) or West Nile virus (WNV), however, amino acid sequences vary^31,32^. NS1 appears to be necessary for ZIKV pathogenesis in the placenta, as a neutralizing antibody to NS1 has shown to limit placental/fetal damage^33^. However, the functional role and mechanism of NS1 in ZIKV pathogenesis remains incompletely understood.

Tunneling nanotubes (TNTs) are actin-based intercellular conduits that extend from and connect to the plasma membrane, measuring 30 to 500 μm in length. TNTs enable the long-range exchange of nucleic acids, proteins, electrical signals (Ca^2+^), lipids, organelles including mitochondria, and infectious particles between connected cells^34–39^. Viruses such as HIV^40,41^, influenza^42^, porcine reproductive and respiratory syndrome virus (PRRSV)^43^, WNV^44^, and SARS-CoV-2^45,46^ have been shown to induce TNTs in infected cells and use these structures to spread to naïve cells. The TNT-associated mode of intercellular transmission likely provides protection for the virus from the extracellular immune response, including neutralizing antibodies, and/or immune cells (as reviewed elsewhere^39,47^).

Here, we report that ZIKV induces the formation of TNTs in multiple cell types, including placental trophoblasts. Among ZIKV structural and non-structural proteins, we demonstrate that NS1 is necessary and sufficient to induce TNT formation. Notably, TNTs were found to be conduits to spread virions, RNA, and proteins from infected to neighboring cells and transfer mitochondria from healthy to ZIKV-NS1-expressing cells. Interactome analysis revealed NS1 is directly or indirectly associated with mitochondrial proteins and pathways leading to mitochondrial transfer. The observed accumulation of mitochondria may provide an energetic boost to virus-infected cells to promote viral replication. Accordingly, disruption of mitochondrial respiration limits virus replication in trophoblast cells. Our findings identify ZIKV-NS1 as the critical mediator for TNT formation, and uncover a previously unrecognized mechanism harnessed by ZIKV to promote intercellular trafficking of the virus and its proteins among placental cells while simultaneously using TNTs to recruit mitochondria directly from neighboring naïve cells. We also show that ZIKV uses TNTs to access and infect these neighboring cells directly without triggering antiviral interferon defenses.

## Results

### ZIKV induces TNT formation in trophoblast cells and transfer viral proteins.

**Figure 1:**
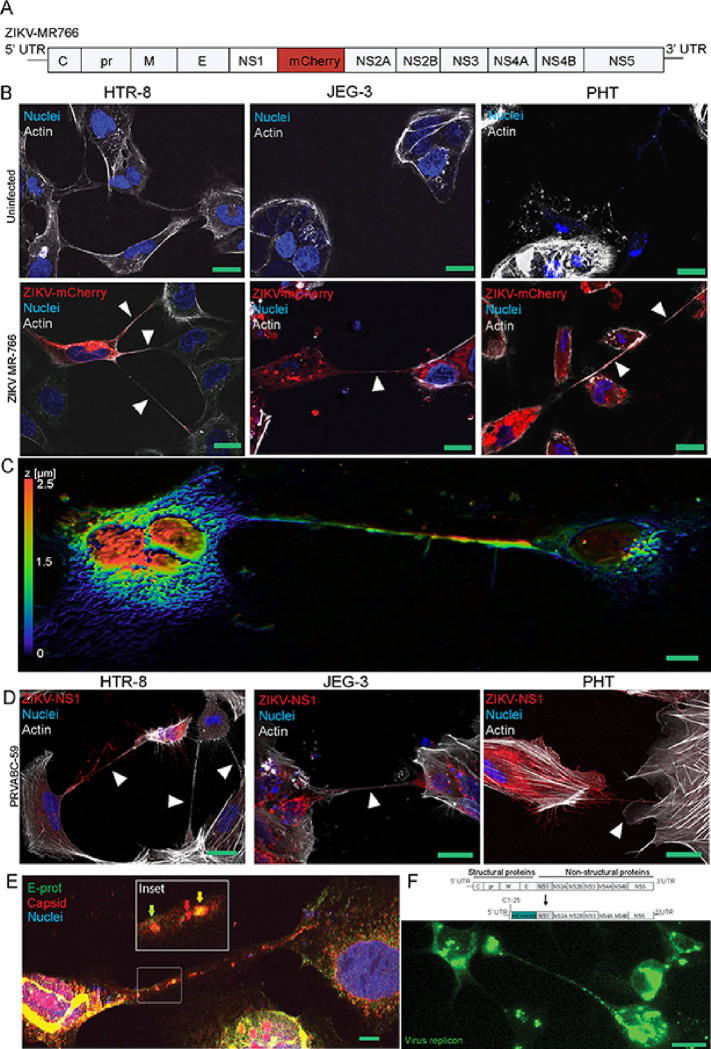
Multiple ZIKV strains induce TNT formation in human placental trophoblast cells and transfer viral proteins. **A)**Schematics showing the generation of fluorescent mCherry-tagged ZIKV MR-766 for live-cell imaging of tunneling nanotubes (TNTs). **B)** ZIKV strain MR-766 induce thin and long TNTs (arrows) connecting infected cells after 24 hours post infection (hpi) in extravillous trophoblast-like cells (HTR-8/SVneo, MOI=0.1) and cytototrophoblasts (JEG-3, MOI=0.1), and primary human trophoblast cells (PHTs, MOI=3) isolated from term placenta. Maximum intensity projection was used to construct z-series images into 2D images (ImageJ). **C)** Maximum intensity projection from a Z-series image of a TNT hovering over the substrate connecting ZIKV-NS1 expressing cells (HTR-8/SVneo). **D)** ZIKV-PRVABC-59 strain also induces TNT formation in HTR-8/SVneo and JEG-3 (MOI=0.1), and PHTs (MOI=3) after 24 hpi. ZIKV NS1 was stained with anti-NS1 and secondary antibodies conjugated to Alexa Fluor 594 dye was used to visualize the protein. **E)** ZIKV MR-766 (MOI=1, 24 hpi) infection of A549 cells induces TNT formation showing colocalization of virus envelope **(E)** and capsid proteins within TNT. ZIKV E and capsid was stained with anti-envelope and anti-capsid and secondary antibodies conjugated to FITC and TRITC was used to visualize the protein. **F)**Schematic showing the construction of ZIKV fluorescent-tagged viral replicon plasmid. Open reading frames (ORFs) for structural proteins capsid, precursor membrane (prM), and E were removed from ZIKV cDNA and replaced with an ORF coding for fluorescent mEmerald protein by site directed mutagenesis and overlap PCR cloning. Live-cell imaging of A549 cells transfected (48 hours) with ZIKV-mEmerald replicon reveals viral RNA and mEmerald within TNTs and neighboring cells. Images B-D were acquired by confocal microscopy at 60X oil objectives lens at 1.4 normal aperture (NA) using a Nikon A1R. Images E and F acquired using a Nikon A1R-MP. Images were processed using the NIS Elements software (Nikon). Nuclei in blue are stained with Hoechst 33342, actin in gray stained with Sir700-Actin Kit, and NS1 in red. Bar = 25 μm.

We engineered ZIKV MR-766 cDNA as previously described^48,49^ to express mCherry using reverse genetics to track real-time infection in live cells (Fig. 1A). ZIKV-mCherry (MR-766) was used to infect trophoblast cells (MOI = 0.1, 24 hours), HTR-8 [extravillous trophoblasts (EVTs)], JEG-3 [cytotrophoblasts (CTBs)], and primary human trophoblast cells (PHTs) isolated from term placentas (Fig. 1B–D). Confocal imaging revealed that ZIKV MR-766 induces the formation of thin and long (> 50 μm) actin-based filaments that hover over the substrate and connect neighboring cells in trophoblast cells (Fig. 1C). These filaments are distinct from intercellular bridges and cytonemes in length (0,2–2 μm in intercellular bridges), function (close-ended extremities) and formation, in addition to being more diverse in composition (actin, anillin, and tubulin)^50,51^. Next, we asked whether TNT induction was a common feature of ZIKV strains and other flaviviruses in the *Flaviviridae* family. Our experiments show in addition to MR-766, the new world ZIKV strain PRVABC-59 was also able to generate TNTs (Fig. 1D). However, no TNTs were observed in trophoblasts infected with dengue virus 2 (DENV2) and yellow fever virus (YFV) (Supplementary Fig. 1), suggesting that TNT formation is not a common feature to all flaviviruses. Together, our findings demonstrate that JEG-3 and HTR-8 (i.e., representing CTBs and EVTs) and PHTs form TNTs in response to ZIKV infection.

Because TNTs are known to transfer a myriad of cargo^38^, we reasoned that ZIKV may co-opt TNTs for viral dissemination. To test this, we infected cells with ZIKV-MR-766 (MOI = 1) and probed with antibodies specific to virus structural proteins envelope (E) and capsid (C) proteins in immunofluorescence assays. In the TNTs formed between neighboring cells, we observed areas of colocalization of capsid and envelope proteins, suggesting that the TNTs might act as a cell-associated conduit for the assembled virions and viral proteins to be trafficked from one cell to another (Fig. 1E).

We next sought to determine if viral genomic RNA can also be transported through TNTs as a mode of viral transmission. We generated a ZIKV MR-766 replicon construct from the cDNA clone, where the virus structural protein genes were replaced with the fluorescent protein mEmerald (Fig. 1F). A549 cells transfected with the replicon construct showed mEmerald expression and formed TNTs. At 48 hours post-transfection (hpt), the neighboring untransfected cells connected via TNTs to transfected cells and showed mEmerald fluorescence, suggesting TNTs allow cell-to-cell transfer of the viral replicon RNA. This observation suggests ZIKV might use TNTs to spread viral genomic RNA from one cell to another even when intact virions are not formed (Fig. 1F). Together, our findings indicate that ZIKV-induced TNT formation serves as a conduit to transfer viral material into uninfected cells.

### The NS1 protein of ZIKV uniquely induces TNT formation.

**Figure 2:**
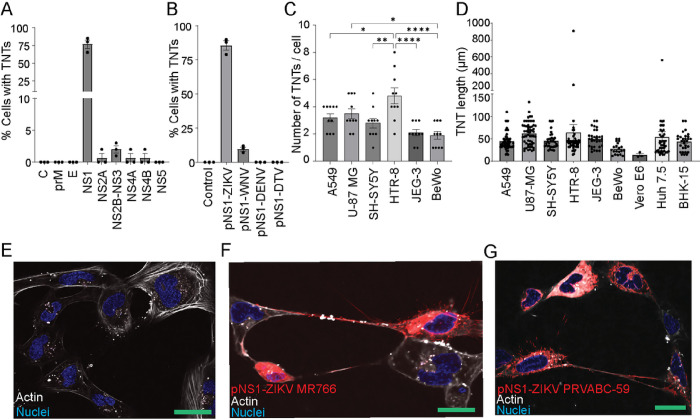
The non-structural protein 1 (NS1) of ZIKV is unique and necessary for inducing TNT formation in multiple cell types. **A**) A549 cells were transfected with fluorescent mCherry-tagged ZIKV proteins and determined their ability to induce TNT formation. **B)** ZIKV-NS1 uniquely induces TNT formation compared to West Nile (WNV)-NS1, dengue (DENV)-NS1, and deer tick virus (DTV)-NS1 in transfected A549 cells. TNT counting was determined by quantifying cells with TNT per 100 cells expressing mCherry-tagged ZIKV proteins after 48 hours post transfection (hpt) and is represented as the average percentage ± standard error (SE), n=3. **C-D**) Expression of NS1 protein induces TNTs. pNS1-ZIKV (MR-766) expression induces TNTs of varying number **C)** and length **D)** in several mammalian cell lines. The number of TNTs per cell and TNT length was quantified and is represented as the average percentage ± SE, n≥ 10 (one-way ANOVA and Dunnett’s post-hoc test, *P≤0.05, **P≤ 0.01, and ****P≤0.0001). **E-G**) Confocal imaging of TNTs formed in HTR-8/SVneo cells. Compared to untransfected cells **E)**, cells transfected NS1 from MR-766 (African strain) **F)** and PRVABC-59 (Asian strain) **G)** induced TNT formation 24 hpt. Images were acquired using a Nikon A1R (60X oil objectives lens at 1.4 normal aperture (NA)). Nuclei in blue are stained with Hoechst 33342, actin in gray stained with Sir700-Actin Kit, and NS1-mCherry is in red. Bar=25μm.

To determine which ZIKV protein is responsible for inducing TNTs in infected cells, we cloned the three structural proteins (C, prM, E) and the seven nonstructural proteins (NS1, NS2A, NS2B-NS3, NS4A, NS4B, and NS5) of ZIKV strain MR-766 with a C-terminal mCherry tag. Transfection of multiple mammalian cells with individual ZIKV proteins revealed that among the viral proteins, NS1 expression uniquely induces TNT formation (Fig. 2A, Supplementary Fig. 1). Despite flavivirus NS1 being structurally well-conserved and being the principal target of positive selection during flavivirus speciation^28,52^, we found that ZIKV-NS1 uniquely induces TNTs compared to NS1 proteins of DENV, and deer tick virus (DTV), although we noted WNV NS1, as described elsewhere^44^, has some limited capacity to form TNTs (Fig. 2B).

TNTs induced in cells by ZIKV NS1 expression ranged in number and length from 15–100 μm (Fig. 2C–D). We noted that HTR-8 cells show increased TNT formation compared to other cell types, including other placental cell types (Fig. 2C). Expression of ZIKV-NS1 from the African (MR-766) and the Asian (PRVABC-59 or ZIKV-NS1^PRVABC^) strains was equally efficient in inducing TNTs in trophoblast cells (Fig. 2E–G) compared to untransfected (Fig. 2E) and recapitulated TNT formation during viral infection (Fig. 1B). Interestingly, while ZIKV-NS1 induces abundant TNT formation in a wide range of mammalian cells, except Vero E6, the restricted ability of WNV-NS1 to form TNTs in neuronal cells (U-87 MG and SH-SY5Y) supports a cell-type and pathogen-specific phenomenon (Supplementary Fig. 2).

### The N-terminus of ZIKV NS1 determines TNT formation.

**Figure 3:**
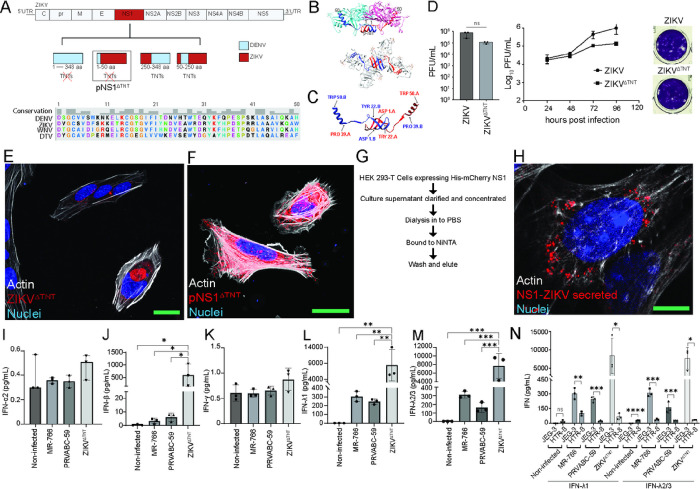
The N-terminus of ZIKV NS1 is necessary to induce TNT formation, and TNTs are functionally important for dampening IFN response. **A)** Schematic depicting plasmid constructs and ZIKV mutants (ZIKV^ΔTNT^) generated in which ZIKV-NS1 sequence was replaced with the nucleotide sequences from dengue virus-2 (DENV2)-NS1 (top panel). The N-terminal 50 amino acids are critical for the TNT-inducing ability of ZIKV-NS1. Alignment of NS1 sequences from DENV2, ZIKV, West Nile virus (WNV), and deer tick virus (DTV) highlighted sequence conservation and potential determinants for TNT formation (bottom panel). Multiple sequence alignment was performed using Clustal Omega and visualized in UCSF Chimera. **B)** Ribbon representation of ZIKV-NS1 dimer structure (PDB:4O6B) where the monomers are colored in teal (chain A) and pink (chain B). The N-terminal 50 amino acids of each monomer are colored in blue and red for chains A and B, respectively. **C)** Ribbon representation of N-terminal 50 amino acids of NS1 dimers (chain A and chain B). Images in B and C were generated using UCSF Chimera **D)** ZIKV^ΔTNT^ retained similar infectious plaque-forming capacity on Vero E6 cells compared to wild-type MR-766 strain as determined by plaque assay (representative image), and viral growth curve (n=3). **E,F)** Confocal imaging to detect TNTs in HTR-8/SVneo cells infected with ZIKV^ΔTNT^ (MOI=0.1) **(E)** and transfected with pNS1^ΔTNT^ **(F)** showing absence of TNTs after 24 hours. G) schematic depicting the methodology for purification and concentration of secreted ZIKV-NS1. **H)** Secreted NS1 does not induce TNT formation. Representative image of HTR-8 cells treated with secreted NS1 for 48 hours showing NS1 in endosomal-like compartments, and no TNT formation. **I-M)** Interferon analysis of ZIKV infected JEG-3 cells. Multiplex assays for interferon type 1/2/3 (LEGENDplex) were performed on supernatants from JEG-3 cells infected with MR-766, PRVABC-59 ZIKV strains, and ZIKV^ΔTNT^ (MOI=0.1) for 48 hrs. **I)** IFN-α2 levels were represented as median ± SE (n=3, Kruskal-Wallis and Dunn’s post-hoc tests). **J)** IFN-β, **K)** IFN-γ, **L)** IFN-λ1, and **M)** IFN-λ2/3 levels were represented as mean ± SD (n=3; ANOVA and Dunnett’s post-hoc test, *P≤0.05, **P≤ 0.01, and ***P≤0.001). **N)** Comparison between JEG-3 and HTR-8’s IFN-λ1 and IFN-λ2/3 response to ZIKV infection (n=3, Student’s t-test, *P≤0.05, **P≤ 0.01, ***P≤0.001, ****P≤0.0001, ns=not significant). Nuclei in blue are stained with Hoechst 33342, actin in gray stained with Sir700-Actin Kit, and NS1 in red. Images **(E, F)** were acquired by confocal microscopy at 60X oil objectives lens at 1.4 normal aperture (NA) using a Nikon A1R and (**H**) A1R-MP. Images were processed using the NIS Elements software (Nikon). Bar= 25 μm.

The NS1 protein consists of three domains: a β-roll dimerization domain (amino acids 1–29) at the N-terminus, a wing domain (amino acids 30–180) with glycosylation sites and subdomains, and a β-ladder domain (amino acids 181–352)^28,32^. To delineate which structural domain of ZIKV-NS1 plays a role in TNT formation, we used site-directed mutagenesis to generate mutant NS1 clones where multiple domains of ZIKV NS1 were substituted with corresponding domains from DENV2-NS1 that did not induce TNTs (Fig. 3A). Subsequent transfection and expression of NS1 mutants revealed that the TNT-forming ability of ZIKV NS1 is determined by the N-terminal 50 amino acids (NS1 mutant pNS1^ΔTNT^) (Fig. 3A), which spans the β-roll dimerization domain and part of the wing domain of NS1 (Fig. 3B–C). Of note, the N-terminal domain of NS1 displays variation across various flaviviruses, with an amino acid identity ranging from 42–44% when compared to ZIKV-NS1 (Fig. 3A, lower panel). Next, to determine that changes introduced in the N-terminal region of NS1 do not affect viral replication and assembly but only affect TNT forming ability, we generated a ZIKV cDNA with mutated NS1 as in pNS1^ΔTNT^ creating a ZIKV^ΔTNT^ mutant (Fig. 3A). Cells infected with ZIKV^ΔTNT^ mutant showed that they are as infectious as the ZIKV wild-type (MR-766) as demonstrated by virus plaque assays and growth kinetic analysis (Fig. 3D), but are unable to form TNTs compared to wild-type ZIKV infection (Fig. 3E), resembling the lack of TNT formation in pNS1^ΔTNT^ expressing cells (Fig. 3F).

Recent work has suggested that NS1 secreted into the extracellular environment influence host responses^53^ and compromises the integrity of the human placenta and endothelial barrier^53,54^. We thus tested whether the secreted NS1 protein could also induce TNTs by treating HTR-8 trophoblast cells with secreted His-mCherry NS1 protein purified from culture supernatants of HEK 293-T cells as described^55^ (Fig. 3G). Confocal images of the His-mCherry NS1-treated cells show accumulation of NS1 in the endosomal vesicles, but no TNTs were formed (Fig. 3H). Together, our data suggests that ZIKV NS1 expression on the plasma membrane, likely as membrane-associated dimers, in necessary for inducing TNTs rather than the NS1 secreted as hexamers.

### TNTs are functionally important for dampening IFN response.

Our results suggest that ZIKV infection promotes TNT formation via the NS1 protein, allowing cell-to-cell transport of virus particles, viral RNA, and proteins. Studies have established that ZIKV infection of trophoblasts is restricted in part due to rapid induction of a robust antiviral response, particularly type III interferons (IFN-lambda or IFN-λ)^16,21,22^. To test the model that ZIKV uses TNTs to traffic to other cells and propagate without triggering an immune response, we performed multiplex assays on supernatants from trophoblast (JEG-3) cells infected with wild type MR-766, or PRVABC-59, or the ZIKV^ΔTNT^ (MOI = 0.1). Remarkably, we found that while the anticipated IFN-β and IFN-λ responses were induced following infection with the MR-766 and PRVABC-59 strains (Fig. 3I–K), the ZIKV^ΔTNT^ mutant incited a remarkable 30–60-fold surge in IFN-λ1, 2, and 3 levels (Fig. 3L–M). These findings provide compelling evidence that the trafficking of ZIKV via TNTs allows ZIKV to elude host antiviral response. Interestingly, we found that HTR-8, which exhibits features of EVTs, appears to have a significantly muted IFN-λ response to ZIKV (MR-766 and PRVABC-59) relative to JEG3 (Fig. 3N), thus implying significantly lower IFN response in HTR-8 cells infected with ZIKV^ΔTNT^ mutant.

### TNT-forming NS1 interacts with mitochondrial proteins.

**Figure 4:**
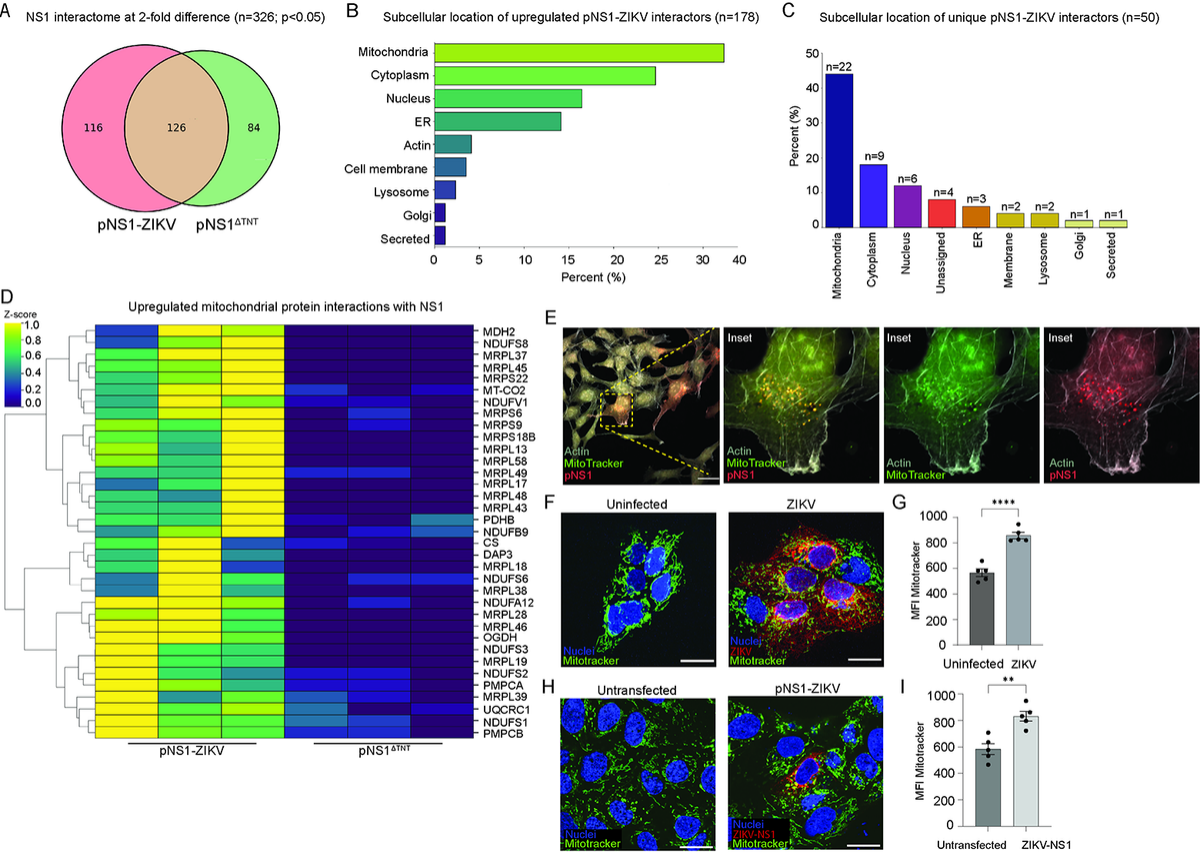
NS1-induced TNTs are associated with mitochondrial proteins. **A-D)** Affinity Purification Mass spectrometry analysis of ZIKV NS1 interacting proteins associated with TNT formation in JEG-3 cells. **A)** Venn diagram showing pNS1-ZIKV and pNS1^ΔTNT^ (non-TNT forming) interacting partners (2-fold difference; P-value ≤0.05) **B)** Subcellular location of 178 proteins enriched with wild-type TNT forming ZIKV NS1 C) unique interacting partners of wild-type TNT forming ZIKV NS1 (n=50) according to the Uniprot database (https://www.uniprot.org/ accessed on 09/29/2023 at 2:28pm). **D)** Heatmap showing differential protein-protein interactions with TNT forming NS1 and mutant NS1^ΔTNT^, n=3. **E)** Representative confocal images showing colocalization of ZIKV-NS1 (red) and mitochondria (green) in HTR-8 trophoblast cells at 16 hours post-transfection (hpt) and co-culture. ROI is magnified as insets and shown as single channels. **F-I)** Mitochondria accumulation in ZIKV infected or ZIKV-NS1 expressing cells. **F)** Representative confocal images showing mitochondria accumulation in JEG-3 infected with ZIKV-mCherry (MR-766) compared to uninfected cells (MOI=0.1, 16 hours post-infection). **G)** comparison of the median fluorescence intensity (MFI) of mitotracker in uninfected and infected cells (n=5, pairwise t-test, ****P≤0.000). **H)** Representative images of JEG-3 cells transfected with pNS1-ZIKV showing mitochondria accumulation and **I**) quantification of mitochondria accumulation via flow cytometry (n=5, Student’s t-test, **P≤ 0.01). Nuclei in blue are stained with Hoechst 33342, mitochondria in green stained with Mitotracker green, and NS1 in red. Images were acquired by confocal microscopy at 40X using a Nikon A1R. Images were processed using the NIS Elements software (Nikon). Bar=10 μm (E) and 25 μm (F,H).

To dissect protein-protein interactions crucial for NS1-mediated TNT formation, we performed affinity-purification mass-spectrometry (AP-MS)^56,57^ using pNS1-ZIKV and pNS1^ΔTNT^ with C-terminal His-tags. We tested the model that any binding partners exclusive to the full-length membrane-bound wild-type NS1 would be necessary for supporting TNT formation. Proteomics data consisting of protein identification and spectral counts were analyzed to estimate relative protein abundance levels^58^ and subsequent identification of unique proteins interacting with TNT-forming ZIKV NS1. Out of the 326 protein-protein interactions with a fold change of > 2 and P < 0.05, 178 protein-protein interactions were upregulated in NS1-ZIKV (Fig. 4A). By querying NS1 protein interactions with the Uniprot database for subcellular compartments, we found that the most represented interactions in trophoblasts with functional TNTs were associated with mitochondrial functions (32%) (Fig. 4B). Next, we investigated the specific protein-protein interactions enriched in TNT-forming ZIKV NS1 (n = 50). Interestingly, we consistently observed that 44% (n = 22/50) of the identified interactions were related to mitochondrial proteins (Fig. 4C), which are highly enriched in ZIKV-NS1 compared to pNS1^ΔTNT^ (Fig. 4D).

### ZIKV-NS1 induces mitochondrial accumulation, and disruption of mitochondria function blocks viral growth.

The prevalence of mitochondrial proteins in the NS1 interactome prompted us to investigate their association with ZIKV infection, NS1, TNT formation, and mitochondrial dysfunction. By co-culturing ZIKV-NS1 expressing cells with untransfected cells pre-stained with Mitotracker green to mark their mitochondria, we found that NS1 displayed colocalization with mitochondria consistent with data obtained from proteomic analysis (Fig. 4E). Next, we investigated the distribution of mitochondria by using confocal microscopy in JEG-3 cells infected with ZIKV (MR-766, MOI = 1, 16 hpi) which revealed increased accumulation of mitochondria in infected cells (Fig. 4F). By co-culturing infected cells with uninfected cells pre-stained with mitotracker green and further analyzing via flow cytometry, we found that infection leads to mitochondria accumulation and that mitochondria were transported from uninfected to infected cells (Fig. 4G). Similarly, ectopic expression of ZIKV NS1 protein in JEG-3 cells transfected with pNS1-ZIKV alone leads to increased mitochondria accumulation as compared to untransfected cells (Fig. 4H), which is further validated by flow cytometry (Fig. 4I).

**Figure 5:**
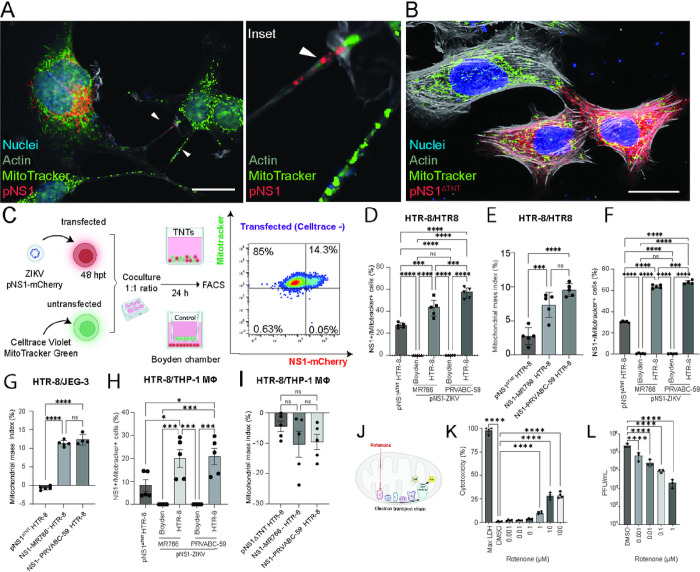
ZIKV NS1 induces mitochondrial transfer between cells via TNTs, and inhibition of mitochondrial activity limits viral replication. **A,B)** Mitochondria transfer through TNTs. Confocal image of HTR-8 cells transfected with pNS1-ZIKV showing mitochondria (green, arrow) and NS1 (red, arrow) transported within F-actin-rich TNTs (gray) (**A**), while cells transfected with pNS1^ΔTNT^ shows limited TNT formation and transfer of mitochondria cargo at 24 hpt (**B**). Images were acquired using a Nikon A1R confocal microscope (40X, 60X oil objectives lens at 1.4 normal aperture (NA)) and processed using the NIS Elements software (Nikon). Nuclei in blue are stained with Hoechst 33342, actin in gray stained with Sir700-Actin Kit, and NS1 in red. Bar= 10 μm (A), 100 μm (B-C). **C-I)** Analysis of mitochondria transfer via TNTs by co-culture experiments. **C)** Experimental set up of co-culture and flow cytometry as in D-I. pNS1-ZIKV transfected cells (NS1-mCherry, acceptor cells) were co-cultured with non-transfected donor cells (MitoTracker green and Celltrace violet) for 24 hours and analyzed by flow cytometry. Experimental control co-cultures were set up in Boyden chambers where donor and acceptor cells are physically separated. Cells were gated singlets, live cells (Live/Dead stain) followed by Celltrace. The resulting cells were gated on NS1-mCherry and Mitotracker expression and represented as the percentage of total acceptor cells (**D, F** and **H**). Graphs showing percentage of NS1-expressing acceptor cells that acquired mitochondria from donor cells in co-culture experiments (**E, G, I**). The homotypic HTR-8/HTR-8 co-cultures **(D,E)** and heterotypic HTR-8/JEG-3 **(F,G)** and heterotypic HTR-8/THP-1 **(H,I)** co-cultures show varying percentage of double positive expressing cells (NS1+ and mitotracker+) and mitochondrial mass index compared to mitotracker and pNS1^ΔTNT^ expressing cells. Quantification of mitochondria transfer was performed using the BD LSRFortessa^™^ cell analyzer, total events collected= 30,000 cells, n= 4–5, Flow cytometry results were analyzed using FlowJo^™^ v10.8 Software (BD Life Sciences). Mitochondrial mass index= 100*((NS1+/Mitotracker+ - NS1−/Mitotracker+) / NS1+/Mitotracker+). **J)** Rotenone is a reversible mitochondrial electron transport chain complex I inhibitor. **K)** Graph showing cytotoxicity of JEG-3 cells to Rotenone 48 hours post-treatment as determined by. Cells were treated with 0.001–0.1 μM of Rotenone and cytotoxicity was determined by lactate dehydrogenase (LDH) cytotoxicity assay, n=4; Brown-Forsythe and Welch ANOVA test. **L)** Rotenone treatment of JEG-3 cells restricts growth of ZIKV MR-766 strain. JEG-3 cells were infected with ZIKV MR766 (MOI=0.1) and treated with different concentrations of Rotenone or DMSO (control) in culture media as shown. At 48 hpi, culture media was harvested, and virus titer determined by plaque assay on Vero-E6 monolayers (n=3). Data was analyzed by one-way ANOVA and post-hoc Dunnett test. ns=non-statistically significant, *P≤0.05, **P≤ 0.01, ***P≤0.001, ****P≤0.0001.

To further investigate the association of ZIKV-NS1 expression and mitochondria accumulation, we co-cultured ZIKV-NS1 expressing HTR-8 cells (acceptor) with homotypic or heterotypic untransfected cells which were pre-stained with Mitotracker green to mark their mitochondria (donor cells). After a 16-hour co-culture, live-cell imaging revealed that NS1 expressing acceptor cells acquired mitochondria from neighboring donor cells via TNTs (Fig. 5A). Consistent with this observation, we further showed that cells expressing pNS1^ΔTNT^ that lack the ability to form TNTs have limited ability to accumulate mitochondria from neighboring donor cells (Fig. 5B). These findings suggest that ZIKV-NS1 mediated TNT formation triggers the transfer of mitochondria from naïve cells to NS1-expressing cells via TNTs.

To quantify the relationship between NS1 expression, TNT formation, and mitochondria accumulation via TNTs, we performed flow cytometry on co-cultured cells (Fig. 5C). Acceptor HTR-8 cells expressing NS1 from ZIKV MR-766 or PRVABC-59 strains were co-cultured with untransfected homotypic (HTR-8), or heterotypic donor cells (JEG-3, THP-1) labeled with Celltrace Violet and Mitotracker Green. Because secreted NS1 can be taken up by untransfected cells (Fig. 3H), we focused on quantifying the impact of ZIKV-NS1 in the donor-to-acceptor transfer of mitochondria. Also, by using THP-1 monocytes differentiated into resting macrophages (M0) and JEG-3 (cytotrophoblasts), we tested the hypothesis that TNTs induced by NS1-expressing cells would mediate mitochondria transfer between different but related cells in the placenta. We also co-cultured donor and acceptor cells in a Boyden chamber, physically separated by a membrane with 400 nm pores allowing secreted signaling molecules to pass through but preventing physical cell-cell contact via TNTs^59^. We did not detect any transfer of mitochondria to NS1-expressing cells in all co-culture settings when physically separated by Boyden chambers (P < 0.0001) (Fig. 5D, F, H). In homotypic HTR-8 cell co-cultures, 99% of acceptor cells acquired mitochondria from donor cells within 24 hours of co-culture (> 99%). Although cells expressing pNS1^ΔTNT^ appear to have obtained mitochondria from donor cells, it was significantly less than the ZIKV-NS1 expressing cells (Fig. 5D–E). We then determined the mitochondrial mass index, which showed an increased percent of mitochondria in ZIKV-NS1 expressing cells relative to non-expressing cells in co-culture, and that ZIKV-NS1 expressing cells showed increased mitochondria transfer compared with pNS1^ΔTNT^ expressing cells (pNS1^ΔTNT^ $\overset{-}{x}=2.7\%$ *vs*. MR-766-NS1 $\overset{-}{x}=7.3\%$ and PRVABC-59-NS1 $\overset{-}{x}=9.5\%$) (Fig. 5E).

We next determined whether ZIKV NS1-TNTs facilitate mitochondria transfer via TNTs in heterotypic co-cultures of HTR-8 acceptor cells and donor JEG-3 cells. Our findings suggest that JEG-3 cells are efficient mitochondria donors and HTR-8 ZIKV-NS1 expressing cells are more effective in siphoning mitochondria (pNS1^ΔTNT^ $\overset{-}{x}=-0.7\%$ *vs*. MR-766-NS1 $\overset{-}{x}=11.2\%$ and PRVABC-59-NS1 $\overset{-}{x}=12.3\%$) (Fig. 5F–G). This finding supports our data showing that HTR-8 makes more TNTs compared with JEG-3 cells (Fig. 2C), which may enable them to transfer mitochondria. We next tested heterotypic transfer between HTR-8 trophoblast cells and THP-1 macrophages and found that HTR-8 could also siphon mitochondria from macrophages albeit at reduced levels relative to that received from other trophoblast cells (Fig. 5H). Remarkably, the resulting mitochondrial mass in acceptor cells expressing NS1 was lower relative to non-expressing cells in co-culture (pNS1^ΔTNT^ $\overset{-}{x}=-4.4\%$ *vs*. MR-766-NS1 $\overset{-}{x}=-10.3\%$ and PRVABC-59-NS1 $\overset{-}{x}=-9.6\%$) (Fig. 5I). Together, these findings underscore that ZIKV-NS1 induced TNTs form cell-to-cell conduits and siphon mitochondria from multiple cell types.

### Inhibition of mitochondrial electron transport affects ZIKV growth.

Viruses are known to manipulate mitochondrial dynamics in infected cells, fostering conditions to facilitate their replication and evade host immune defenses^60,61^. To determine if the acquisition of mitochondria is pivotal for ZIKV replication or propagation, we treated JEG-3 cells with Rotenone, a well-documented inhibitor of mitochondrial complex I (Fig. 5J) and determined its effect on ZIKV growth. Using a LDH assay, we identified that Rotenone concentrations ranging between 0.001 to 0.1 μM were non-toxic to JEG-3 cells (Fig. 5K) whereas HTR-8 cells exhibited cytotoxicity at Rotenone concentration of 0.01 μM, suggesting that HTR-8 cells are more sensitive to mitochondrial dysfunction than JEG-3 cells (data not shown). We next evaluated the impact of concentrations from 0.001 to 1 μM of Rotenone on ZIKV replication in JEG-3 cells. Remarkably, Rotenone caused a significant reduction in virus production within JEG-3 trophoblast cells at 0.001 μM as evidenced from plaque assays (Fig. 5L). These findings underscore that mitochondrial transfer and intact mitochondrial function is integral for ZIKV production.

## Discussion

Our investigation reveals a previously unknown mechanism exploited by ZIKV, setting it apart from other flaviviruses such as WNV, DTV, YFV, and DENV as it possesses the capacity to induce TNT formation via its NS1 protein in multiple cell types, including trophoblasts. A limited number of viruses have been reported to induce the formation of TNTs in infected cells, such as HIV, HSV and IAV (reviewed in ^39^). TNTs have also been shown to transfer various cargos, including virus particles, viral RNA, and replication complexes between connected cells^38,39,45^. Our results indicated that by inducing the formation of TNTs, ZIKV gains direct entry into neighboring cells, leading to a rapid cell-associated spread of ZIKV, potentially increasing the pathogenicity. Notably, TNTs provide a conduit for the higher ZIKV transmissibility through cell-to-cell interactions compared to cell-free virus transmission, as neutralizing antibodies are ineffective in inhibiting viral spread *in vitro*^62^. Other positive-strand RNA viruses such as SARS-CoV-2 and CHIKV have been shown to exploit cell-to-cell transmission to infect non-permissive cells that lack viral entry factors and bypass the effect of neutralizing antibodies and important blood tissue barriers^45,63^.

We found that among flaviviruses (DENV, WNV, DTV) the first 50 amino acids at the N-terminal region of NS1 confer ZIKV its unique ability to induce TNTs. The mechanism by which the amino acids 1–50 of ZIKV NS1 induce TNT formation is not understood and is beyond the scope of this manuscript. Previous studies show N-terminal residues forming the β-roll and connector subdomain of the wing are important for membrane binding^64^, but vary between flaviviruses. We speculate that the unique property of ZIKV NS1 to induce TNT formation is linked to this varying amino acid sequence and the resulting variation in charge distribution and membrane binding properties. We provide evidence that membrane-bound NS1 is likely necessary for TNT formation since secreted NS1 is unable to induce TNTs. Interestingly, WNV-NS1 has been shown to induce formation of TNT-like structures, while secreted NS1 promotes remodeling of the cytoskeleton suggestive of F-actin depolymerization in a cell type specific manner^44^. Given that high levels of the extracellular NS1 hexamers circulate in the bloodstream of flavivirus infected patients^65^, it is unlikely that secreted ZIKV NS1 induce TNTs but rather leads to tissue permeability and endothelial damage^53,54,66,67^. Interestingly, anti-NS1 monoclonal antibodies (mAbs) targeting cell-surface NS1 (presumably expressed as a dimer^28^) or the N-terminal region of NS1 have been shown to limit ZIKV infection in animal models^33,68–70^. While the protective mechanisms of non-neutralizing ZIKV NS1-targeted mAbs remain yet to be elucidated, a growing body of evidence shows that NS1-based vaccines confer protection against ZIKV infection in animal models^71,72,72–75^. Further investigation is needed to determine whether mAbs or NS1-based vaccines target TNT formation in ZIKV-infected cells, potentially limiting viral infection and spread.

We report that TNTs triggered by ZIKV infection transport viral material and weaken the IFN response of the host. The host immune defense against ZIKV initially involves the recognition of viral RNA by receptors like RIG-I, which interact with the mitochondrial antiviral signaling protein (MAVS) leading to the production of type I and III interferons, such as IFN lambda, which activate antiviral genes^76^. IFN-lambda is constitutively expressed by primary human trophoblasts and is known to limit ZIKV infection^16^. Infection of JEG-3 cells, which are representative of CTBs, with ZIKV^ΔTNT^ which do not drive TNT formation, it elicits multi-fold increase in IFN response compared to ZIKV wild-type infection (TNT-competent). Previous studies have shown that ZIKV dampens host IFN and RIG-I-dependent innate immunity and manipulates mitochondrial dynamics^23,27,77,78^. Importantly, since mutant ZIKV^ΔTNT^ is fully replication competent, infectious and had similar growth patterns compared to wild-type ZIKV, we propose that the TNT forming capacity is the key differential resulting in immune IFN response evasion by ZIKV While we cannot rule out the influence of DENV-NS1 N-terminal region on IFN signaling, the surge of IFN response to ZIKV^ΔTNT^ suggests that TNTs are shielding the virus from the immune system. Our discovery that TNTs are co-opted by ZIKV to dampen trophoblast IFN response, facilitating the transfer of viral particles and mitochondria highlights a previously unknown function of NS1. We speculate that inhibition of TNT formation and halting mitochondria siphoning from neighboring cells could result in more robust RIG-I and MDA5 signaling and limit viral transmission.

Our work sheds light on the specificity of the N-terminal region of ZIKV-NS1 in inducing TNTs and mitochondria accumulation via TNTs. While mitochondria transfer between cells through TNTs has been documented in various physiological and pathological conditions, its role in viral infections remains incompletely understood^39,79,80^. Nonetheless, PRRSV has been shown to promote mitochondria transfer from uninfected to infected cells in allogeneic and xenogeneic co-cultures to rescue infected cells from apoptosis/necrosis. Also, PRRSV proteins were found colocalized with mitochondria in TNTs, suggesting that the virus hitchhikes with mitochondria for intercellular transportation^43,80^, consistent with our findings that ZIKV NS1 protein-protein interactions were highly enriched for mitochondrial proteins, and NS1 colocalizes with mitochondria acquired from neighboring cells. TNT formation has been reported to be associated with ROS expression levels and the release of ‘call-for-help signals’ such as S100 proteins^80–82^.

Of note, the intercellular exchange of mitochondria via TNTs has been shown as a mechanism to rescue stressed cells^83–85^ by providing metabolic support and delaying cell death^59,80^. Our study showing mitochondria accumulation in trophoblasts infected with ZIKV via transfer of mitochondria via TNTs from naïve neighboring cells suggests that ZIKV co-opts a physiological stress response to benefit its own transmission and survival. We discovered that preemptive Rotenone treatment inhibits viral replication, suggesting that ZIKV requires functional mitochondria for replication. Interestingly, we found that even at nanomolar concentrations, Rotenone induces cytotoxicity in HTR-8 cells. The lower tolerance to mitochondrial damage in HTR-8 cells could potentially translate into a greater need for mitochondria and thus promoting increased formation of TNTs to siphon mitochondria from different cell types.

ZIKV have been noted to infect multiple placental cell compartments including fetal endothelial cells, cytotrophoblasts, and fetal macrophages, but not clearly in STBs^15,16,18,86,87^, the trophoblast cell layer contacting the maternal blood that protects the fetus from blood-borne infections and constitutively secrets type III IFN^16,88^. Furthermore, it has been shown that the maternal decidual compartment with the tolerogenic HLA-G + EVTs^89^ important for mediating immune tolerance of the semiallogeneic fetus typically produce basal levels of IFN^90,91^ and remain susceptible to ZIKV infection^87,92^. Given that cell-cell interactions between fetal and maternal cells occurs at the anchoring villi, which are specialized structures that attach and anchor the placenta to the decidua, the formation of TNTs by ZIKV in EVTs could contribute at least in part for its dissemination from the decidua to the placenta. Accordingly, we found that HTR-8 trophoblast cells which are EVT-like, are more responsive to TNT formation upon ZIKV infection and ZIKV-NS1 expression compared to other trophoblast cells (CTBs and STBs/PHTs) and secrete only limited levels of IFN. Our data suggests that HTR-8/EVTs which harbor higher levels of ZIKV, lower lFN response and more abundant TNT formation, and increased cargo transfer between homotypic and heterotypic cells could spread ZIKV more efficiently to less permissive cells. Recently, TNTs extending up to 7 μm connecting endothelial cells have been demonstrated in human term placenta using volume electron microscopy^93,94^. Further, TNTs have been observed in co-culture of primary human decidual immune cells and trophoblast cells in placental organ-on-chip models^95^. The functional relevance of TNTs in maternal and fetal cell-cell interactions during infections is further demonstrated by decidual NK cell immune response to *Listeria monocytogenes* infection of trophoblast cells^96^. Further investigation of other viruses known to infect the placenta and able to induce TNT formation, such as SARS-CoV-2 and HIV is warranted^41,45,97,98^. It is intriguing to suppose that TNT formation may serve as the defining feature of placenta-infecting pathogens.

Our study offers a new mechanism for ZIKV to infect placental cells and involving NS1-mitochondria interactions and offers vital insights into developing therapeutic strategies against this stealth transmission mode.

## Methods

### Cell lines

Human trophoblasts cells Bewo (ATCC, Cat. CCL-98), JEG-3 (ATCC, Cat. HTB-36), HTR-8 (ATCC, Cat. CRL-3271), and PHTs were cultured with Dulbecco’s Modified Eagle Medium/Nutrient Mixture F-12, (DMEM/F-12 GIBCO, Cat. 11330032) supplemented with 10% fetal bovine serum (FBS) (Gibco, 16140071) and maintained at 37 °C with 5% CO2. THP-1 (ATCC, Cat. TIB-202) was cultured in Roswell Park Memorial Institute (RPMI) 1640 Medium (Gibco, A1049101) supplemented with 10% FBS and 0.05 mM 2-Mercaptoethanol (Gibco, 21985023). THP-1 differentiation was performed as previously described^99^. PHTs were isolated from term placentas from deidentified uncomplicated pregnancies at Barnes-Jewish Hospital Labor and Delivery Service, St. Louis, MO. PHTs were thawed and cultured in a 6-well plate (Corning, 353046) at 2 × 10^6^ cells/well using cell culture media described previously^100^. Following cell attachment, PHTs were washed with Iscove’s Modified Dulbecco’s Medium (IMDM; Gibco, 12440053) with 10% FBS, 100 U/mL Penicillin-Streptomycin (Gibco, 15070063) 10 μM Y-27632 (Selleckchem, S1049), 0.05 mM 2-Mercaptoethanol (Gibco, 21985023), and 30 ng/mL mouse EGF Recombinant Protein (Gibco, PMG8041). Cells were maintained in IMDM media for three days, subcultured at a 1:2 split ratio, and maintained for 5 days until virus infection in IMDM medium without Y-27632. HEK-293T (ATCC, Cat. CRL-1573), Vero-E6 (ATCC, Cat. CRL-1586), A549 (Cat. CCL-185), Huh 7.5 (a kind gift from Dr. Charles M. Rice, Rockefeller University), BHK-15 (a kind gift from Dr. Richard J. Kuhn, Purdue University), U-87 MG (ATCC, Cat. HTB-14), and SH-SY5Y (ATCC, Cat. CRL-2266) were cultured in Dulbecco’s Modified Eagle’s Medium (DMEM, Gibco, 12800–082) supplemented with 10% FBS, non-essential amino acids (NEAA, HyClone, SH30238.01), and Penicillin-Streptomycin (PS, Corning, 30-002-CI) and maintained at 37 °C with 5% CO_2_. C636 cells were maintained in Minimum Essential Medium (MEM, GIBCO, #41500–018) supplemented with 10% FBS and Penicillin-Streptomycin (PS, Corning, 30-002-CI) and maintained at 30 °C with 5% CO_2_.

### ZIKV strains, cDNAs, and expression plasmids.

The prototypical African ZIKV MR-766 Uganda strain, and PRVABC-59 were obtained from BEI resources. The cDNA clone derived from the 1947 Uganda MR-766 ZIKV genome placed at the transcriptional initiation site of the cytomegalovirus (CMV) promoter^49^ was modified to replace Venus tag with mCherry for this study. Plasmids expressing individual ZIKV proteins and NS1 proteins from ZIKV MR-766 (pNS1-ZIKV), Dengue Virus-2 (pNS1-DENV2), West Nile Virus NY99 strain (pNS1-WNV), and Powassan virus lineage II (Deer Tick Virus) (pNS1-DTV) were generated by PCR amplification using Q5DNA Polymerase (NEB#M0492) and cloning into pCDNA3.1 or Ligation Independent Cloning into pcDNA3 mCherry LIC cloning vector (Addgene, #30125). Amino acid substitutions were introduced into ZIKV cDNA clones and expression plasmids by site-directed mutagenesis using Phusion DNA polymerase (NEB, E0553S), followed by DpnI digestion and transformation into NEB Stable Competent *E. coli* (New England BioLabs Inc., C3040H). Plasmids were obtained from overnight cultures of *E. coli* colonies grown in Luria Bertani medium using the Qiagen miniprep kit (Qiagen, 27104) or Qiagen midiprep kit (Qiagen, 12143), and sequences of the resulting clones were confirmed via Sanger sequencing at The Sequencing Core Facility at The Pennsylvania State University. DNAs were quantified using NanoDrop^™^ One (ThermoFisher Scientific, Waltham, MA, USA), and aliquots stored at −20 C.

To generate mutant ZIKV, cDNAs were transfected into HEK 293T cells using Lipofectamine 2000 (ThermoFisher, Cat #11668030). After 12h, the media was replaced and incubated at 37 C under 5% CO2. Cell culture supernatants were collected after 4 days of growth, filtered with a 0.45 μm mixed cellulose membrane (MCE) filter, added HEPES at a final concentration of 10 mM, and stored at −80°C. For expression of individual ZIKV proteins and flavivirus NS1 proteins, plasmids were transfected into cell lines of interest using Lipofectamine 2000. All experiments were performed under biosafety level 2 (BSL2) conditions.

### ZIKA virus titration and propagation

ZIKV was propagated in African green monkey kidney (Vero) cells (ATCC, Cat. CCL-81) cultured in DMEM/F-12 (Dulbecco’s Modified Eagle Medium/Nutrient Mixture F-12, GIBCO, Cat. 11330032) supplemented with 10% FBS (Fetal Bovine Serum, GIBCO, Cat. 16140071). Confluent cells were infected with ZIKV in DMEM/F-12 2% FBS for five days, the supernatant containing viruses was harvested, and aliquots were stored at −80 C. Virus titers were determined by plaque assay of serial dilutions on Vero-E6 monolayers as described previously^11,101^.

### Affinity purification-mass spectrometry

The affinity purification-mass spectrometry method was employed to purify His-tagged NS1 proteins using Ni-NTA resin, as previously reported^56,57^. We generated two plasmids expressing C terminal Octa-histidine-tagged wild type NS1 and the mutant pNS1-ZIKV^ΔTNT^ by site-directed mutagenesis. Plasmids were purified using a Qiagen Midiprep kit, sanger sequenced, and used to transfect cells grown in 150 mm dishes using Lipofectamine 2000. Briefly, the pNS1-ZIKV and pNS1^ΔTNT^ plasmids were transfected into JEG-3 cells. At 48 hpt, cells were washed, and harvested in 1 ml PBS supplemented with Protease Inhibitor Cocktail (Millipore Sigma, P8340. The cells were lysed by sonication using a microtip attached to a sonicator (Branson), and membrane fractions were purified by ultracentrifugation at 100,000 g for 90 minutes using a TLA 120.2 rotor and Optima TLX centrifuge (Beckman Coulter). The membrane pelleted obtained after centrifugation was resuspended in 1 ml PBS supplemented with Protease Inhibitor Cocktail and 1% Fos-choline 12 and incubated at 4°C with gentle rocking for 2 h. The extracted proteins were separated from insoluble fraction by a second round of ultracentrifugation and allowed to bind to NiNTA resin for 30 min at 4°C with gentle rocking. The resin was washed with 10 column volumes of PBS with 0.01% Foscholine-12 (Anatrace, F308) followed by 2 column volumes of PBS buffer and subjected to mass spectrometry analysis at the Indiana University Proteomics Core, Indianapolis, USA. Beads were briefly resuspended in 8 M Urea, 100 mM Tris pH 8.5. Cysteines were then reduced with 5 mM tris(2-carboxyethyl)phosphine hydrochloride (TCEP), and alkylated with 10 mM chloroacetamide (CAM). Samples were diluted to less than 2 M Urea with 50 mM Tris pH 8.5 and digested overnight at 37°C with 0.5 μg Mass Spectrometry Grade Trypsin/Lys-C Mix (cat. num. V5072, Promega). Samples were filtered and acidified with formic acid (FA) before LC/MS/MS.

### Nano-LC-MS/MS analysis

Nano-LC-MS/MS analyses were performed on a Q Exactive^™^ Plus Hybrid Quadrupole-Orbitrap^™^ Mass Spectrometer coupled to an UltiMate 3000 UHPLC RSLCnano System (Thermo Fisher Scientific, Hanna-Bremen, Germany). Approximately 10 μL from each sample was loaded and concentrated using an Acclaim^™^ PepMap^™^ 100 trap column (Cat. num. 164535, Thermo Fisher Scientific) at 3 μL/min for 5 mins in 100% Buffer A (Water + 0.1% FA). Chromatographic separation was done on an Easy-Spray PepMap column (Cat. num. ES901, Thermo Fisher Scientific, ID 75 μm, 15 cm length, 3 μm particles with 100 Å pore sizes). The LC gradient consisted of holding at 3% Buffer B (Acetonitrile + 0.1% FA) for 5 minutes, followed by a gradient from 3–35% Buffer B over 75 minutes, followed by an increase to 95% Buffer B for 2 minutes, a decrease to 3% Buffer B for 2 minutes and hold at 3% Buffer B for 2 minutes.

The mass spectrometer was operated in positive mode, with a spray voltage of 1.8 kV and ion transfer capillary temperature of 250°C. Data-dependent acquisition with the top 15 most intense ions for MS/MS. Full MS scan parameters were: Resolution 70k, AGC target 3E6, m/z range 200–2000; MS2 parameters were: Resolution 17.5k; AGC target 1E5; Maximum IT 50 ms; Isolation window 4.0 m/z; Fixed first mass 100 m/z; NCE 30.0.

### Mass spectrometry data analysis

The resulting RAW files from mass spectrometry experiments were analyzed using Proteome DiscoverÔ 2.5 (Thermo Fisher Scientific). The MS/MS spectra were searched against a database containing reviewed *Homo sapiens* proteins (downloaded from the UniProt on 10/04/2019) plus common contaminants. SEQUEST HT search engine was used with trypsin as the proteolytic enzyme, including two allowed missed cleavages, precursor mass tolerance of 10 ppm; and a fragment mass tolerance of 0.2 Da. Static modification of carbamidomethylation on cysteine residues was included, as well as dynamic or variable modifications of oxidation on methionine, peptide N-terminal methionine loss, acetylation, and methionine loss+N-terminal acetylation. Fixed PSM Validator was used as an FDR filter, and results were loaded into Scaffold^™^ 4 (Proteome Software) to visualize data and calculate Fisher’s Exact test p-values with Benjamini-Hochburg correction. Relative quantitation of proteins comparing NS1 binding experiments used either Scaffold quantitation functions or Normalized Spectral Abundance Factor calculations and accounted for related isoform identifications^58,102^. The STRING application (Version 2.1.0)^103^ within Cytoscape (version 3.9.1)^104^ was used for visualization of functional enrichment based on protein interaction networks from observed proteins. Subcellular localization was determined from the UniProt database (Release 2023_3).

### Immunofluorescence assay

Cells were grown on glass coverslips in 24 well plates and were infected with ZIKV at an MOI of 0.01. At 36 hours post-transfection, cells were fixed with 3.7% paraformaldehyde in PBS for 15 minutes and further permeabilized with 0.1% Triton X-100 in PBS for 5 minutes at room temperature. Cells were blocked with 10 mg/ml bovine serum albumin (BSA, Sigma-Aldrich, A7906) in PBS overnight at 4°C. Subsequently, the blocking buffer was removed, and cells were treated with primary antibodies against Envelope protein (4G2, a kind gift from Theodore C. Pearson), capsid protein (GeneTex, GTX134186), or NS1 protein (GeneTex, GTX133323) followed by treatment with fluorescein isothiocyanate (FITC), tetramethyl rhodamine isothiocyanate (TRITC), or Alexa Fluor 594 dye-conjugated secondary antibodies for 2 hours. Nuclei were stained using Hoechst-33342 (Invitrogen, H3570) according to the manufacturer’s instructions. Cells were washed 3 times with PBS, and coverslips were mounted onto microscope slides with FluorSave Reagent (Calbiochem, 3457) and confocal images were acquired using a Nikon A1R-MP confocal microscope fitted with a 60x oil objective lens with 1.4 numerical aperture (NA) and processed using the NIS Elements software (Nikon). Brightness and contrast were adjusted using look up tables (LUTs).

For imaging mitochondria transfer via TNTs, transfected or infected cells were co-cultured for 24 hours with cells pre-stained with 500 nM Mitotracker^™^ Green (Invitrogen, Cat. M7514). Samples were gently fixed in cold Phosphate-buffered saline (PBS) containing 3.5% formaldehyde methanol-free (ThermoFisher Scientific, Cat. 47392) and 0.5% glutaraldehyde (Polysciences, Cat. BLI1909) and stained with Hoechst 33342 (Invitrogen, Cat. H3570) and SiR700-Actin Kit (Cytoskeleton; CY-SC013). The specimens were prepared using mounting media (Polyscience, 18606–100) following standard protocols and imaged using the ECLIPSE Ti2 inverted microscope (Nikon).

### Live-cell imaging

For screening TNT formation, cells were grown in 4-well chamber slides (IBIDI, #80427) and transfected with expression plasmids, or infected with ZIKV and ZIKV TNT- and incubated for 48 hours. Live imaging was performed in a Nikon A1R-MP confocal microscope, using a heated 60× oil immersion objective (1.4 NA) in a live imaging chamber (Tokai Hit, Fujinomiya, Shizuoka Prefecture, Japan) supplied with 5% CO2 at 37°C. Images and videos were acquired using NIS-Elements software. TNTs were determined based on z-stack image acquisition and 3D reconstruction of maximum intensity projection.

For testing virus transfer, live-cell imaging was performed as described previously with the following modifications^105,106^. Cells plated on a 4-chamber borosilicate cover glass (Fischer Scientific Pittsburgh, PA) at 25% confluence will be infected with fluorescent protein tagged-ZIKV or transfected with plasmids expressing fluorescently tagged proteins. The media was replaced with Opti-MEM I reduced-serum medium (Invitrogen) and stained with Hoechst 33342 stain (nucleus) and Vybrant DiD cell-labeling solution (membrane and vesicles) in conjunction with fluorescent protein-tagged viruses.

### Quantification of mitochondria transfer

Transfer of mitochondria transfer via TNTs was quantified by flow cytometry. The day before, HTR-8 or JEG-3 cells were seeded in a 6-well plate at a density of 500,000 cells/well (Corning, 353046). Untransfected cells were stained 4 hours before co-culture with 150 nM MitoTracker Green FM (Invitrogen; M7514) and 8 μM CellTrace Violet (Invitrogen, C34557). Cells were washed twice with complete medium after staining and again before co-culture to remove any potential unbound dye. HTR-8 cells were transfected with 1 μg of NS1 plasmid (ZIKV, DENV, DTV) using Lipofectamine^™^ 3000 Transfection Reagent (Invitrogen, L3000001). At 4 hours of post-transfection (hpt), 250,000 transfected and untransfected cells were harvested and co-cultured with homotypic or heterotypic cells at a 1:1 ratio with or without Boyden chambers (Falcon, 353090). After 24 hours post-co-culture, cells were harvested, stained with viability dye (Invitrogen, L10119), and analyzed in the BD LSRFortessa (Becton Dickinson). For THP-1 co-culture, 250,000 cells/well (Thermo Scientific, 174901) were seeded and primed with 150 nM Phorbol 12-myristate 13-acetate (PMA, Sigma, P8139) for 24 hours and maintained in culture for three days until fully adherent and differentiated before co-culture with transfected HTR-8. Positive and negative gates were set based on FMO controls. Cells were gated for singlet, followed by live cells (Live/Dead stain) and CellTrace (Supplementary Fig. 3). The resulting cells were evaluated for NS1-mCherry and Mitotracker expression—total events = 30,000 cells.

### Quantification of mitochondria accumulation

Mitochondria accumulation was quantified by flow cytometry. JEG-3 were seeded at 250,000 cells/well and cultured overnight. On the next day, uninfected cells were stained 4 hours before co-culture with 150 nM MitoTracker Green FM (Invitrogen, M7514) and 8 μM CellTrace Violet (Invitrogen, C34557). JEG-3 were either inoculated with ZIKV-mCherry (MOI=0.1) or mock infected for 2 hours and immediately co-cultured with uninfected cells at 1:1 ratio for 24 hours. For mitochondria accumulation in transfected cells, JEG-3 were either transfected with 1 μg of pNS1-ZIKV or no plasmid using Lipofectamine^™^ 3000 Transfection Reagent (Invitrogen, L3000001). At 4 hpt, transfected cells were co-cultured with untransfected cells at 1:1 ratio for 24 hours. At 24 hours post-co-culture, cells were harvested, stained with viability dye (Invitrogen, L10119), and analyzed on BD LSRFortessa (Becton Dickinson). Cells were gated for singlet, followed by live (Live/Dead stain) and CellTrace (CellTrace Violet). The resulting cells were evaluated for median fluorescence intensity of MitoTracker Green to quantify accumulation of mitochondria in infected/ transfected cells. Total events = 30,000 live cells.

### Interferon level analysis

JEG-3 cells were infected with ZIKV including MR-766, PRVABC-59, and ZIKV^ΔTNT^ (MOI=0.1) for 48 hours. LEGENDplex^™^ Human Type 1/2/3 Interferon Panel (5-plex) (BioLegend, 740350) was used to measure IFN-α2, IFN-β, IFN-γ, IFN-λ1, and IFN-λ2/3 levels simultaneously on the infected cells’ supernatant according to the manufacturer’s instructions. Bead-bound cytokines were measured using High Throughput Sampler (HTS) option on BD LSR III (Becton Dickinson) and concentrations were calculated using the LEGENDplex^™^ Data Analysis Software (BioLegend).

### Lactate dehydrogenase (LDH) cytotoxicity assay

LDH release was measured using CytoTox 96^®^ Non-Radioactive Cytotoxicity Assay (Promega, G1780) according to the manufacturer’s instructions. Briefly, cells were seeded in 96-well plate at density of 1 × 10^4^ cells/well. After overnight culture, cells were treated with Rotenone (Sigma, R8875) ranging from 0.001 to 100 μM for 24 hours. Cell culture media were collected and used for measuring LDH release. LDH levels was determined by recording absorbance at 490 nm using BioTek Epoch Microplate Spectrophotometer and the percent cytotoxicity was calculated per manufacturer’s instructions: Percent cytotoxicity = 100 × (Experimental LDH Release/ Maximum LDH Release).

### Statistical analysis and reproducibility

All analyses were carried out using GraphPad Prism 9. No statistical methods were used to predetermine sample sizes. The normal distribution of continuous variables was assessed by the Shapiro-Wilk test and the statistical significance of pairwise comparisons were assessed by the Student’s t-test or the Mann-Whitney test when appropriate. Comparisons of three of more groups were performed by the one-way ANOVA followed by the Tukey’s test to determine differences between groups. Where data showed a nonparametric distribution, a Kruskal–Wallis test was used followed by post-hoc Dunn’s test. Descriptive statistics, statistical tests, and post-hoc tests for multiple comparisons are reported in each figure legends and the accompanying source data. For comparison of mitochondria transfer, five independent experiments were used. For quantification of TNTs, five randomly chosen microscope fields containing ZIKV-NS1 expressing cells from three biologically independent replicates were used for statistical analysis. For all comparisons, a two-sided P value < 0.05 was considered statistically significant.

## Data Availability

Further information and requests for resources and reagents should be directed to and will be fulfilled by the lead contacts, Indira Mysorekar (Indira.Mysorekar@bcm.edu) or Anoop Narayanan (aun84@psu.edu).
